# Right Ventricular Volumes and Function normalized to body surface area, age and sex in a large cohort of well-treated Thalassemia Major without myocardial iron overload

**DOI:** 10.1186/1532-429X-13-S1-P299

**Published:** 2011-02-02

**Authors:** Antonella Meloni, Maria Chiara Dell'Amico, Brunella Favilli, Giovanni D Aquaro, Ait-Ali Lamia, Elisabetta Chiodi, Claudio Ascioti, Stefania Renne, Pier Paolo Bitti, Calogera Gerardi, Vincenzo Positano, Massimo Lombardi, Alessia Pepe

**Affiliations:** 1 “G. Monasterio” Foundation and Institute of Clinical Physiology, CNR, Pisa, Italy; 2 Arcispedale “S. Anna”, Ferrara, Italy; 3 P.O. “Giovanni Paolo II”, Lamezia Terme, Italy; 4 P. O. San Francesco. ASL Nuoro, Nuoro, Italy; 5 Ospedale "Sant'Elia", Caltanisetta, Italy

## Introduction

Cardiovascular Magnetic Resonance CMR has provided the opportunity to quantify right ventricular (RV) parameters with excellent reproducibility and accuracy. The role of the RV is gaining ground in thalassemia major (TM) patients and this population could experience different “normal” RV values due to chronic anemia and eventually pre-existing iron burdens. In literature, there are no data on the ranges for “normal” RV volumes and ejection fraction (EF) in TM patients. The aim of this study was to establish the ranges for normal RV volumes, mass and ejection fraction normalized to the influence of body surface area (BSA), age and sex from CMR in a large cohort of well-treated TM patients without myocardial iron overload.

## Materials

Among the 923 TM patients enrolled in the Myocardial Iron Overload (MIOT) network who underwent CMR for the assessment of cardiac iron overload, function and fibrosis, we selected 142 patients with no known risk factors or history of cardiac disease, normal electrocardiogram, no myocardial iron overload (all the cardiac segments with a normal T2* value) and no myocardial fibrosis. All patients had been regularly transfused and chelated since early childhood. Moreover, we studied 71 healthy subjects matched for age and sex. RV function parameters were quantitatively evaluated in a standard way by SSFP cine images using MASS® software. RV end-diastolic volume (EDV), end-systolic volume (ESV) and stroke volume (SV) were normalized by body surface area (EDVI, ESVI, SVI).

## Results

Table 1 shows the comparison of the CMR parameters with differentiation for sex and age in TM patients and healthy subjects and the cut-off of normality defined as mean - 2 standard deviation (SD). TM patients showed significantly lower BSA than the controls (P<0.0001). TM males (except age group 14-20 yrs) showed significantly higher RV EF compared to controls. In TM patients all LV volumes indexes were significantly larger in males than in females (P<0.0001 in all age groups). The EF was not different between the sexes. In males as well as in females the RV volumes were no significant different among the age groups, while in males the EF was significant different (P=0.004).

**Table 1:** 

	< 14			14-20			20-30			30-40			>=40		
	**TM**	**H**	**P**	**TM**	**H**	**P**	**TM**	**H**	**P**	**TM**	**H**	**P**	**TM**	**H**	**P**

**Males**	*N=7*	*N=7*		*N=6*	*N=6*		*N=25*	*N=15*		*N=23*	*N=11*		*N=6*	*N=5*	

EDVI (ml/m^2^)	88 ±16 (56)	80 ± 12	0.309	89 ± 16 (57)	89 ± 17	0.946	96 ± 21 (54)	103 ± 17	0.245	91 ± 16 (59)	82 ± 13	0.139	90 ± 13 (64)	81 ± 9	0.228
ESVI (ml/m^2^)	26 ± 3 (20)	31 ± 8	0.273	36 ± 7 (22)	35 ± 9	0.931	34 ± 8 (18)	44 ± 12	0.0004	33 ± 8 (17)	33 ± 8	0.952	28 ± 6 (16)	35 ± 3	0.045
SVI (ml/m^2^)	61 ± 16 (29)	49 ± 6	0.108	53 ± 11 (31)	54 ± 10	0.836	61 ± 18 (25)	60 ± 9	0.882	58 ± 11 (36)	46 ± 17	0.010	62 ± 8 (46)	46 ± 8	0.007
EF (%)	68 ± 6 (56)	62 ± 5	0.048	59 ± 5 (49)	61 ± 5	0.579	64 ± 4 (56)	58 ± 6	0.001	64 ± 5 (54)	61 ± 7	0.189	69 ± 3 (63)	57 ± 5	<0.0001

**Females**	*N=2*	*N=2*	*N=2*		*N=8*	*N=6*		*N=24*	*N=6*		*N=33*	*N=8*		*N=8*	*N=5*

EDVI (ml/m^2^)	53 ± 16 (21)	61 ± 1	0.587	77 ± 8 (61)	79 ± 9	0.866	78 ± 14 (50)	78 ± 12	0.990	73 ± 13 (47)	77 ± 9	0.427	74 ± 11 (52)	70 ± 16	0.648
ESVI (ml/m^2^)	18 ± 1 (17)	20 ±**4**	0.656	29 ± 5	30 ± 5	0.777	28 ± 8 (12)	31 ± 9	0.378	24 ± 7	26 ± 6	0.317	26 ± 5 (16)	25 ± 12	0.922
SVI (ml/m^2^)	35 ± 5 (25)	41 ± 3	0.635	47 ± 4 (39)	49 ± 7	0.673	51 ± 9 (33)	47 ± 7	0.384	49 ± 8 (30)	51 ± 10	0.738	47 ± 6 (35)	45 ± 5	0.517
EF (%)	64 ± 9 (46)	68 ± 6	0.687	62 ± 3 (56)	62 ± 6	0.997	65 ± 6 (53)	61 ± 7	0.171	68 ± 5 (58)	66 ± 8	0.329	67 ± 7 (55)	66 ± 8	0.862

## Conclusion

In a large cohort of well-treated TM patients males showed significantly higher RV EF compared to controls. Appropriate “normal” reference ranges normalized to BSA, sex and age should be used to avoid misdiagnosis of cardiomiopathy in the clinical arena in TM patients.

